# Human, Oceanographic and Habitat Drivers of Central and Western Pacific Coral Reef Fish Assemblages

**DOI:** 10.1371/journal.pone.0120516

**Published:** 2015-04-01

**Authors:** Ivor D. Williams, Julia K. Baum, Adel Heenan, Katharine M. Hanson, Marc O. Nadon, Russell E. Brainard

**Affiliations:** 1 Coral Reef Ecosystem Division, Pacific Islands Fisheries Science Center, National Oceanographic and Atmospheric Administration, Honolulu, Hawaii, United States of America; 2 Department of Biology, University of Victoria, Victoria, British Columbia, Canada; 3 Joint Institute for Marine and Atmospheric Research, University of Hawaii at Manoa, Honolulu, Hawaii, United States of America; 4 Department of Oceanography, School of Ocean and Earth Science and Technology, University of Hawaii at Manoa, Honolulu, Hawaii, United States of America; 5 Fisheries Research and Monitoring Division, Pacific Islands Fisheries Science Center, National Oceanographic and Atmospheric Administration, Honolulu, Hawaii, United States of America; Leibniz Center for Tropical Marine Ecology, GERMANY

## Abstract

Coral reefs around US- and US-affiliated Pacific islands and atolls span wide oceanographic gradients and levels of human impact. Here we examine the relative influence of these factors on coral reef fish biomass, using data from a consistent large-scale ecosystem monitoring program conducted by scientific divers over the course of >2,000 hours of underwater observation at 1,934 sites, across ~40 islands and atolls. Consistent with previous smaller-scale studies, our results show sharp declines in reef fish biomass at relatively low human population density, followed by more gradual declines as human population density increased further. Adjusting for other factors, the highest levels of oceanic productivity among our study locations were associated with more than double the biomass of reef fishes (including ~4 times the biomass of planktivores and piscivores) compared to islands with lowest oceanic productivity. Our results emphasize that coral reef areas do not all have equal ability to sustain large reef fish stocks, and that what is natural varies significantly amongst locations. Comparisons of biomass estimates derived from visual surveys with predicted biomass in the absence of humans indicated that total reef fish biomass was depleted by 61% to 69% at populated islands in the Mariana Archipelago; by 20% to 78% in the Main Hawaiian islands; and by 21% to 56% in American Samoa.

## Introduction

Coral reefs are frequently the focus of scientific, media, and conservation attention because of their vulnerability to human impacts, but there remain a number of extremely isolated and uninhabited coral reef areas in the Indian and Pacific Oceans. Because those places are in many cases several hundred kilometres or more from the nearest human populations, biological communities at those isolated reef ecosystems have been used as baselines against which to assess the extent of human impacts to reef ecosystems around human-populated islands. Much of that work has focused on reef fish assemblages and there is now abundant evidence that, compared to human-populated islands, remote coral reefs typically have three to four times or more standing biomass of reef fishes, with relatively large portions of biomass in upper trophic levels and made up of larger-bodied taxa [1–4].

Extremely remote and minimally-disturbed coral reef areas are almost certainly better exemplars of natural reef ecosystems than possible alternatives, such as long established marine protected areas (MPAs) around populated islands, because protection is often incomplete or compliance uncertain within MPAs [5], because some reef fish species have ranges that are considerably larger than the size of the areas protected within most MPAs [4], and because remote reefs are isolated from nearly all human impacts, not just fishing. However, it is also important to recognize that among islands and regions there are substantial differences in reef habitats and structure that are likely independent of human impacts [6], as well as in potentially influential oceanic factors such as wave energy, water temperature, and oceanic productivity [7] that confound our ability to understand what might be considered ‘natural’ for a particular region or reef. For example, estimated baseline densities for Pacific reef sharks varied by a factor of >4 among island groups in the Pacific, with much of that difference apparently due to differences in oceanic productivity and therefore presumed increased productivity of reef sharks’ prey base [8].

Here, we use survey data collected along wide gradients of environmental conditions and potential human impact to investigate drivers of differences in reef fish assemblages among ~40 US and US-affiliated islands and atolls spread widely across the western and central Pacific. The availability of consistent large-scale survey data, together with recently-synthesized satellite-derived environmental data from the same locations [7] allowed us to use generalized modelling and multi-model inference to assess the strength of associations between a range of factors—human population density, wave-energy, oceanic productivity, temperature, coral cover and structural complexity—and the standing biomass of reef fishes, as well as of particular reef fish trophic groupings. Additionally, recognizing the high degree of natural variability in factors driving differences among coral reef areas, we use models to predict ranges of baseline reef fish biomass at each reef area in the absence of humans, and use those predictions to generate estimates of reef fish depletion at human-populated islands.

## Methods

### Ethics Statement

Partner agencies that contributed to field work and survey operations and provided permissions to work in local waters include the Papahānaumokuākea Marine National Monument, the US Fish and Wildlife Service, the Department of the Interior, the State of Hawaii Department of Land and Natural Resources, the Commonwealth of the Northern Mariana Islands Papahānaumokuākea Marine National Monument, US Fish and Wildlife Service, State of Hawaii Department of Land and Natural Resources, Commonwealth of the Northern Mariana Islands (CNMI) Division of Fish and Wildlife, CNMI Coastal Resources Management Office, CNMI Division of Environmental Quality, Guam Division of Aquatic and Wildlife Resources, and the American Samoa Department of Marine and Wildlife Resources.

### Study Region and Survey Program

**Fig 1:**
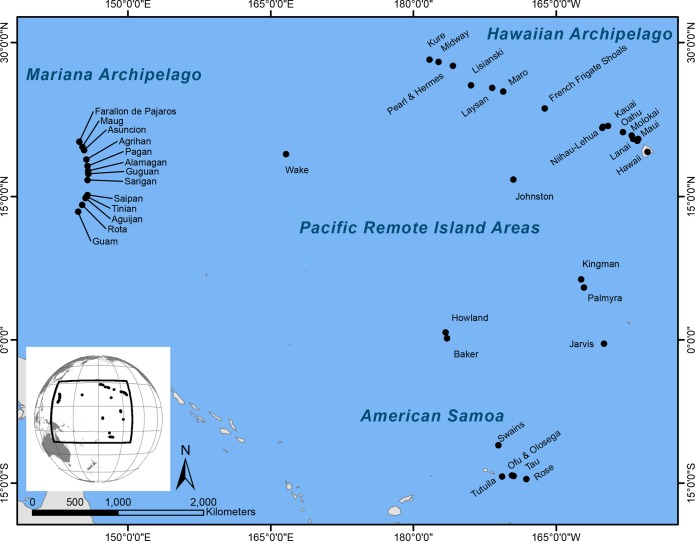
Locations of reef areas (i.e. islands and atolls) included in this study. Fish and habitat data used in this study comes from surveys conducted between 2010 and 2013.

We analyzed co-located fish and benthic data collected between 2010 and 2013 as part of the Pacific Reef Assessment and Monitoring Program (Pacific RAMP), which is conducted by NOAA’s Pacific Islands Fisheries Science Center, Coral Reef Ecosystem Division (CRED) and partners. Pacific RAMP involves multidisciplinary monitoring of shallow water (<30m) coral reef habitats at US and US-affiliated islands and atolls (henceforth ‘reef areas’) in the central and western Pacific: specifically, the Hawaiian and Mariana Archipelagos, the Pacific Remote Island Areas (Johnston and Wake Atolls, and the US Line and Phoenix Islands) and American Samoa (Fig. 1). Survey locations range from heavily populated and somewhat urbanized (e.g. Oahu, Maui, Guam, and Tutuila) to extremely remote and uninhabited reef areas several hundred kilometers or more from the nearest human population centers.

The great majority of surveys were conducted during 1–3 month long Pacific RAMP cruises, with surveys conducted by trained scientific divers working off small boats. Typically, each reef area was visited for 3–5 days, allowing for ~ 20–50 sites surveyed per each reef area per cruise. Additional site surveys came from supplemental survey efforts using the same protocols, survey design, and largely involving a consistent core group of survey divers.

At each reef area, the target domain was hardbottom in <30 m depth, stratified by reef-zone (forereef; backreef; lagoon) and depth bin (0–6 m; 6–18 m; 18–30 m). To maximize comparability, only data from forereef surveys were used for this study, which comprised 79% of the hardbottom habitat and 89% of the sites surveyed. At each survey location, primary and backup survey sites were selected prior to the survey visit, using a formal randomization procedure, and based on the habitat, reef-zone and bathymetric maps developed by CRED.

### Survey Methods

Details of the survey protocol are given in [9], but in brief: on reaching a target survey site, a pair of divers entered the water and swam directly down to the reef, where one of the divers laid a 30 m gray Dacron transect line along a depth contour. Divers surveyed fishes using a modification of the paired stationary point count (SPC) method developed for multi-agency reef fish monitoring in the Florida Keys [10]. For the SPC, the 2 divers conducted simultaneous counts in adjacent visually estimated 15 m diameter cylinders extending from the substrate to the limits of vertical visibility. Each SPC consisted of two components: a 5-minute species enumeration period in which divers recorded all species present in or moving through their cylinder, followed by a tallying portion, in which divers systematically recorded the number and size (total length to nearest cm) of all fishes of each taxon on their list. The tallying portion was conducted as a series of rapid visual sweeps, with one species grouping counted per sweep. The divers’ goal was to get a near instantaneous record of fishes present within their cylinder. Divers also recorded a range of other ‘non-instantaneous’ data on fish assemblages, but we restricted this analysis to the instantaneous counts that are likely the best measures of fish standing stock, as non-instantaneous counts tend to overcount mobile species [11].

On completing the fish count, divers estimated benthic cover (% cover per functional group, including hard coral) and structural complexity within the SPC cylinders. For all surveys, data from the two adjacent cylinders were pooled into a mean value for the site.

### Predictor Variables

We collated and synthesized data on a range of environmental, habitat and anthropogenic covariates known to influence coral reef fish assemblages [3,8,12–16] (Table 1). Remote-sensing data on sea-surface temperature (SST), chlorophyll-a (CHL), and derived wave-energy were taken from a recent study which reported long-term (9–25 year) masked and quality-controlled averages of those variables for the islands and atolls in the US Pacific regions that are the focus of this study [7]. SST and CHL data come from 4-km resolution pixels surrounding each island. For SST, data are averaged from all non-land pixels intersecting or within the 30 m contour of each island or atoll. However, for CHL, pixels that intersected or were contained within the 30 m contour were masked to remove spurious data points associated with shallow-bottom reflectance, and so CHL values for each island come from the innermost band of 4-km pixels outside of the 30 m contour [7]. CHL values are therefore a proxy for primary productivity of pelagic waters surrounding reef areas, which we henceforth refer to as ‘oceanic productivity’. Mean wave energy (WV) was derived from information on wave height, period, and dominant direction, taken from NOAA’s Wave Watch III model (http://polar.ncep.noaha.gov/waves). Following [8] we used mean SST from the coldest month of each year, i.e. the lower climatological mean (SSTL) as our temperature covariate (n.b. long-term mean SSTL was very highly correlated with mean SST at the islands in that study, Pearson’s r = 0.96).

**Table 1:** Island-scale predictor variables.

Predictor	Description
SSTL	Lower climatological mean of sea surface temperature, i.e. the average of mean temperature in the coldest month of each year between 1985 and 2009
CHL	Climatological mean of Chlorophyll-a between July 2002-and May2011
WV	Climatological mean of wave energy between 1997 and 2010
HC	% Hard Coral
CX	Structural complexity (mean substrate height within fish-count cylinders)
HUM	Square root transformed human population resident on an island divided by area of forereef
HDIST	Square root transformed ‘distant’ human population per area of forereef.- distant human population is number of people living within 200km of survey sites but not resident on the island
AT	Atoll (Y/N?)—is reef area an atoll or bank (Y) or not (N)

Values represent island-means: CHL, WV and SSTL were obtained from satellite-derived sources [7] and represent long-term (9–25 yr.) averages of oceanic waters surrounding. HC and CX are visually-estimated by divers during fish surveys. Human population data per island comes from the 2010 US census (http://www.census.gov/2010census/). Area of <30m hard-bottom habitat per island comes from CRED GIS maps used for survey design, and collated from a range of internal and external sources. Island-scale values calculated as weighted averages of all sites within an island. Island-scale weighting of sites described in the Methods section.

Mean island-wide hard-coral cover (HC) and structural complexity (CX) come from visual estimates made by divers during surveys. Structural complexity was measured in two ways: in 2010 and 2011 divers estimated complexity on a 5-point scale (1 = very low, to 5 = very high); but in 2012 and 2013 the method was revised and divers instead estimated the maximum vertical relief within their cylinder and the proportion of their cylinder in different relief bins (<0.2 m; 0.2–0.5 m; 0.5–1.0 m; 1.0–1.5 m; >1.5 m from substrate) [17]. Those data were then used to generate a mean vertical relief value for each cylinder. The two *in-situ* complexity measures were calibrated using data from reef areas where we had both types of data (S1 Fig.), to allow us to generate a single average of measured or calibrated mean vertical substrate height as a metric of complexity (CX).

Reliable and comparable socioeconomic data, including coral reef fishery data, are not available for our study locations [18], and we have therefore used human population density scaled per unit reef area as a consistent metric of potential human impact. Human population data come from the 2010 US census (http://www.census.gov/2010census/). We used two measures of human population: (i) local human population density, i.e. humans resident at an island divided by reef area (HUM) and (ii) distant human population density, i.e. total humans living within 200 km but not resident on the island, again divided by reef area (HDIST). We included distant human population as a covariate in recognition of the growing body of evidence that long-range factors such as proximity to larger human populations and markets can generally be expected to have large impacts on local reef fish assemblages [19,20]. The standard reef-area measure used was the amount of hardbottom forereef habitat in <30 m, i.e. the forereef component of the domain of the survey program—and was derived from the habitat and bathymetric GIS maps maintained by CRED and collated from a range of internal and external sources. We also included one factorial variable, namely whether the island was an atoll or not (AT). Values of island-scale explanatory variables are shown in Table 2 and S1 Table.

**Table 2:** Study Island and Explanatory Variables.

REGION	ISLAND	LATITUDE	LONGITUDE	ATOLL	Forereef Area (Ha)	Human Population	Distant Human Pop.	CHL(mg m^-3^)	WV(kW m^-1^)	SSTL (°C)
N. Mariana	Farallon de Pajaros	20.542	144.895		138	-	-	0.04	21.00	25.09
	Maug	20.023	145.222		314	-	-	0.04	19.55	25.46
	Asuncion	19.693	145.401		249	-	-	0.04	19.55	25.58
	Agrihan	18.775	145.668		851	-	-	0.04	21.82	25.97
	Pagan	18.105	145.760		1,513	-	-	0.05	20.33	26.09
	Sarigan-Guguan-Alamagan	17.249	145.816		267	-	15,080	0.04	20.24	26.27
S. Mariana	Saipan	15.191	145.735		3,539	48,220	21,336	0.05	18.05	26.72
	Tinian	15.025	145.627		1,414	3,136	173,919	0.04	18.05	26.75
	Aguijan	14.850	145.553		406	-	212,599	0.04	18.36	26.81
	Rota	14.149	145.193		1,331	2,527	210,714	0.03	16.89	26.84
	Guam	13.451	144.773		7,296	162,810	10,785	0.04	15.54	26.98
NWHI	Kure	28.413	-178.339	X	2,438	-	-	0.09	45.51	18.98
	Midway	28.235	-177.367	X	3,294	40	-	0.09	45.80	19.31
	Pearl & Hermes	27.876	-175.799	X	8,498	-	-	0.12	46.10	19.69
	Lisianski	26.016	-173.941		30,955	-	-	0.10	42.01	21.33
	Laysan	25.773	-171.730		3,400	-	-	0.08	42.26	21.53
	Maro	25.401	-170.557	X	25,607	-	-	0.10	40.56	21.73
	French Frigate	23.767	-166.228	X	8,873	-	-	0.09	39.17	22.78
MHI	Kauai	22.037	-159.568		18,127	65,689	665,687	0.09	34.81	23.56
	Niihau	21.917	-160.157		9,266	170	68,021	0.07	34.81	23.65
	Oahu	21.471	-157.956		25,119	953,207	199,685	0.08	28.56	23.87
	Molokai	21.102	-157.058		12,730	7,404	1,147,664	0.09	28.56	23.89
	Lanai	20.850	-156.900		3,004	3,102	1,182,900	0.08	28.56	24.10
	Maui	20.834	-156.369		11,122	144,444	930,397	0.08	29.73	23.85
	Hawaii	19.683	-155.548		16,840	185,079	96,224	0.07	26.48	24.12
PRIA	Wake	19.299	166.625	X	280	150	-	0.04	28.03	25.39
	Johnston	16.746	-169.510	X	713	-	-	0.05	34.03	25.04
	Kingman	6.405	-162.405	X	351	-	-	0.13	30.32	27.37
	Palmyra	5.877	-162.092	X	2,793	20	-	0.12	30.32	27.30
	Howland	0.807	-176.620		173	-	-	0.18	21.49	27.34
	Baker	0.194	-176.474		390	-	-	0.19	20.85	27.46
	Jarvis	-0.374	-159.993		366	-	-	0.21	24.90	26.52
Am. Samoa	Swains	-11.056	-171.080		281	17	-	0.05	21.15	27.91
	Ofu & Olosega	-14.172	-169.649		1,055	353	55,149	0.04	23.40	27.11
	Tau	-14.240	-169.464		1,003	790	54,712	0.04	23.40	27.15
	Tutuila	-14.295	-170.681		4,888	55,149	353	0.06	22.34	27.12
	Rose	-14.543	-168.156	X	110	-	1,143	0.04	27.12	26.94

NB Values for Sarigan-Guguan-Alamagan are means of island-scale values for these three small adjacent islands.

### Data Analysis

In total, we analyzed data from 1,934 sites on 37 reef areas, constituting > 2,000 hours of underwater observation. Following our survey design and standard procedure when reporting data from Pacific RAMP, we pooled data from three adjacent small Northern Mariana Islands—Sarigan, Guguan, Alamagan into a single reporting unit “SG&A” and consider that as a single reef area. We excluded three Northwestern Hawaiian Island (NWHI) reef areas from the analysis. Gardner Pinnacles was not included because it was not covered by the study we acquired oceanographic data from [7]. We excluded Nihoa and Necker Islands because of low sample size and low island-scale data precision—both having only 8 survey sites (lowest at other island being 12), and coefficients of variation (standard error/mean) of total fish biomass of 87% and 47% respectively (highest at any other island being 35%, mean of other islands 14%). We also excluded the 48 sites at inhabited islands that were within marine protected areas with strictly enforced controls on fishing, because the prohibition of fishing could confound broader relationships between human population density and fish assemblages in those areas.

The core response measure used for this study was estimated biomass of fishes per unit area (hereafter “biomass”). The mass of individual fishes was calculated using length to weight conversion parameters taken from published and web-based sources [21,22]. Data were pooled into ‘all fishes’ and into four trophic groupings based on diet information taken largely from FishBase [21]: “primary consumers” (herbivores and detritivores); ‘secondary consumers’ (omnivores and benthic invertivores); ‘planktivores’; and ‘piscivores’ (http://escholarship.org/uc/item/5394f7m3). We excluded all sharks and jacks from biomass estimates because of our concern that different responses of those highly mobile roving piscivores to the presence of divers could be a source of bias among study locations. Most obviously, the ‘mobbing’ behavior of jacks and sharks appears to cause substantial overestimation of their densities in small-scale diver surveys in the Northwestern Hawaiian Islands [23,24].

Site-level reef fish biomass, hard coral cover, and complexity values were turned into island-scale averages by first calculating within depth-strata mean and variance and then calculating weighted mean and variance using the formulas given in [10] with strata weighted by their respective sizes, i.e. area of hardbottom habitat in that strata.

### Data Exploration and Modeling

Prior to developing analytical models, we conducted data exploration following the recommendations of [25]. Because untransformed local and distant human population per reef area predictors, HUM and HDIST, were extremely right-skewed, we square root transformed those variables. We examined potential colinearity among predictor variables using Pearson correlations and variance inflation factors. Wave energy and sea surface temperature metrics (WV, SSTL) were strongly negatively correlated (r = -0.88), but other correlations were not problematic—the next highest correlation coefficients being between wave energy and the factor Atoll (WV and AT, r = 0.61) and between complexity and hard coral cover (CX and HC, r = 0.41). We therefore determined not to include both WV and SSTL in single models, and calculated variance inflation factors (VIF) for the two potential subsets of predictor variables (all other than SSTL; all other than WV). The highest VIF for any predictor in either subset was 2.1-for WV—lower than our *a priori* cut-off value of 3 [25].

We used generalized additive models (GAM) to examine relationships between inter-island mean fish biomass and inter-island variations in the predictors using the *mgcv* packages in R [26]. Models were fitted with a gamma distribution using a log link. Given our relatively small number of response values, we limited the number of knots to five to prevent overfitting.

We used model selection and model-averaging procedures from the *MuMIn* R package v1.9.13 [27] based on Akaike’s Information Criterion corrected for small sample sizes (AICc) [28]. For each response variable, we first ran all possible models with the predictor data set (with SSTL and WV never included in the same model) using the procedure *dredge*. We retained all models with Akaike weight > 0.05. The ‘variable importance’ output from *dredge* gives sum total weight of all models containing a particular variable. As total model weights sum to 1, high values of variable importance are indicative of predictor variables that occur in a large portion of highly-ranked models. Using the retained models, we then used the *model*.*avg* procedure to generate a weighted model average suitable for model prediction using the *predict* function. We predicted total fish biomass in the absence of humans—i.e. the complete dataset, but with HUM and HDIST set to 0 for all islands, as in Nadon et al. [8].

To ease interpretation of relationships between the response and predictor variables, we used the *predict* procedure to generate visualizations of smoothers in the top-ranked models, setting the predictor variable of interest to values equally spaced between that variable’s min and max, and setting all other predictor variables to their means. We used the resulting output to generate smoothers with scaled biomass response shown against untransformed predictor variables.

All analyses were conducted using R version 3.0.3 [29]. Analysis scripts are provided in the supplementary materials (S1 Text).

## Results

The grand mean of total reef fish biomass, excluding reef sharks and jacks, across all reef areas was 40.5 g m^-2^ (SD 27.8 g m^-2^). Total fish biomass tended to be lower at islands with large human populations—the lowest biomass value (mean ± SE) being 8.3 ± 0.7 g m^-2^ at Oahu in the Hawaiian Islands—and highest at unpopulated and remote islands, particularly those in the US Line Islands—highest biomass being 129.6 ± 18.3 g m^-2^ at Kingman Reef (S1 Table). ‘Primary consumers’, primarily made up of surgeonfishes and parrotfishes, were the largest component of biomass averaged across all islands, with a grand mean of 16.8 g m^-2^ (SD 9.6 g m^-2^, S1 Table). Grand means for other consumer groups ranged from 7.0 g m^-2^ (SD 3.8 g m^-2^) to 8.4 g m^-2^ (SD 9.8 g m^-2^, S1 Table).

Mean reef-area hard coral cover ranged from 2.9 ± 1.8% at Midway (NWHI) to 38.4 ± 3.0% at Swains (American Samoa) with a grand mean of 21.0% (SD 9.1%). Structural complexity (mean substrate height) ranged from 0.29 ± 0.02 m at Oahu to 0.92 ± 0.09 m at Swains.

### Reef Fish Biomass Models

**Fig 2:**
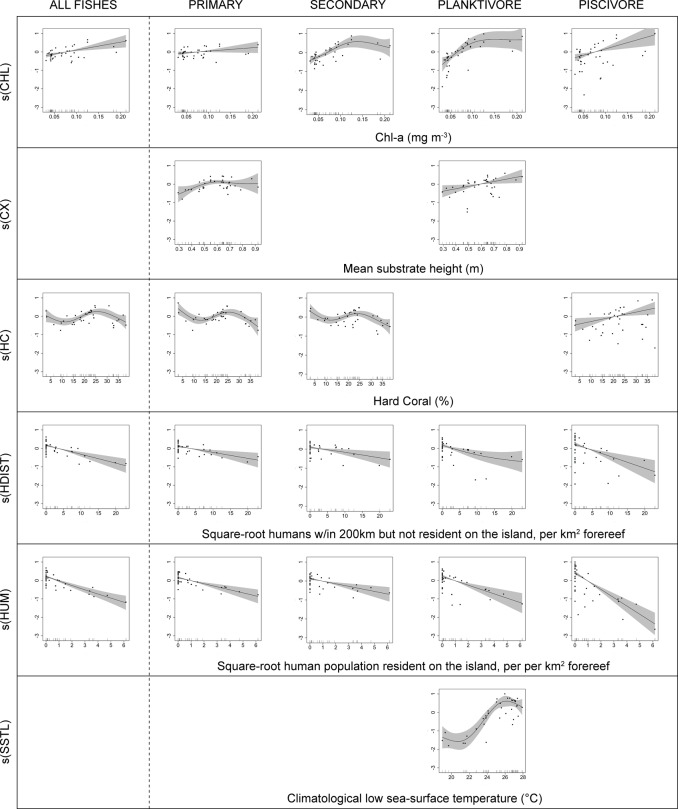
Smoothers of predictor variables retained in highest ranked models of ‘all fishes’ and of consumer groups. Shaded areas show 95% confidence.

For ‘all fishes’, all models with Akaike weight > 0.05 included Chlorophyll-a (CHL), both local and distant human population density (HUM & HDIST), and hard-coral cover (HC) (Table 3). Model smoothers for the top-ranked model show that total reef fish biomass was positively correlated with CHL, and negatively with both human population variables (Fig. 2). The relationship with hard coral cover indicated reef fish biomass peaks at very low cover and at intermediate values of around 25% (Fig. 2). Other retained models, apart from the model with those variables plus SSTL, had ~1 degree of freedom more than the top model and **Δ**AICc values of > 2 (Table 3), indicating that the additional variables in those models were uninformative parameters [30]. The model with SSTL had ~3 degrees of freedom more than the top-ranked model, and **Δ**AICc of ~3, so the addition of SSTL improved model fit, even if not sufficiently to overcome the penalty for additional complexity. Variable importance (vi) for the 4 predictors in the top-ranked model was 0.990 or greater for CHL, HDIST and HUM, and 0.852 for HC; and no other predictor variable had vi > 0.256 (Table 3).

**Table 3:** Best GAMs (all models with weight > 0.05).

Model Terms							Model Support					
AT	CHL	CX	HC	HDIST	HUM	SSTL^1^	WV^1^	d.f.	Adj-R^2^	AICc	Δ AICc	Weight
ALL FISHES excluding sharks and jacks												
	X		X	X	X			8.5	0.833	288.46	0.00	0.383
X	X		X	X	X			9.3	0.835	290.97	2.52	0.109
	X	X	X	X	X			9.4	0.834	291.19	2.74	0.097
	X		X	X	X	X		11.4	0.866	291.55	3.09	0.081
	X		X	X	X		X	10.0	0.841	292.01	3.55	0.065
**0.212**	**0.990**	**0.256**	**0.852**	**0.993**	**1.000**	**0.219**	**0.114**	←**variable importance sum wt.→**				**0.735**
PRIMARY CONSUMERS												
	X	X	X	X	X			10.6	0.829	224.45	0.00	0.105
		X	X	X	X			10.9	0.834	224.56	0.11	0.099
X		X	X	X	X			10.5	0.826	224.80	0.35	0.088
		X	X	X	X		X	10.7	0.829	224.80	0.35	0.088
X			X	X	X			8.6	0.786	225.04	0.59	0.078
X	X	X	X	X	X			11.3	0.839	225.14	0.69	0.074
			X	X	X		X	8.6	0.784	225.29	0.84	0.069
			X	X	X			7.6	0.762	225.34	0.90	0.067
X	X		X	X	X			9.7	0.805	225.44	0.99	0.064
**0.420**	**0.413**	**0.585**	**0.998**	**0.982**	**0.999**	**0.095**	**0.275**					**0.730**
SECONDARY CONSUMERS												
	X		X	X	X			9.8	0.730	168.39	0.00	0.183
X	X		X	X	X			11.0	0.756	169.66	1.27	0.097
	X		X		X			8.1	0.670	169.68	1.29	0.096
	X				X			4.9	0.552	170.98	2.59	0.050
**0.261**	**0.996**	**0.190**	**0.753**	**0.598**	**0.948**	**0.134**	**0.190**					**0.426**
PLANKTIVORES												
X	X	X		X	X	X		12.3	0.907	180.84	0.00	0.379
	X			X	X	X		10.4	0.879	181.96	1.12	0.217
X	X			X	X	X		11.0	0.885	182.98	2.14	0.130
X	X			X	X		X	9.0	0.854	183.78	2.94	0.087
X	X	X		X	X		X	9.4	0.857	184.53	3.68	0.060
**0.756**	**0.993**	**0.526**	**0.090**	**0.982**	**0.998**	**0.820**	**0.180**					**0.873**
PISCIVORES excluding sharks and jacks												
	X		X	X	X		6.0	0.818	184.11	0.00	0.307
	X		X	X	X	X		7.4	0.824	186.88	2.77	0.077
	X		X	X	X		X	7.3	0.823	187.12	3.01	0.068
	X	X	X	X	X		7.0	0.818	187.17	3.06	0.067
**0.204**	**0.997**	**0.213**	**0.690**	**0.920**	**1.000**	**0.223**	**0.165**					**0.519**

Details of model terms are given in Table 1: AT = Atoll(y/n); CHL = oceanic chlorophyll; CX = complexity; HC = hard coral; HDIST and HUM are distant and local human population; SSTL-sea surface temperature climatological low; and WV = wave energy. Note weights and variable importance are calculated from all models.

(1) WV and SSTL are never included in same model.

### Impacts of Predictor Variables

**Fig 3:**
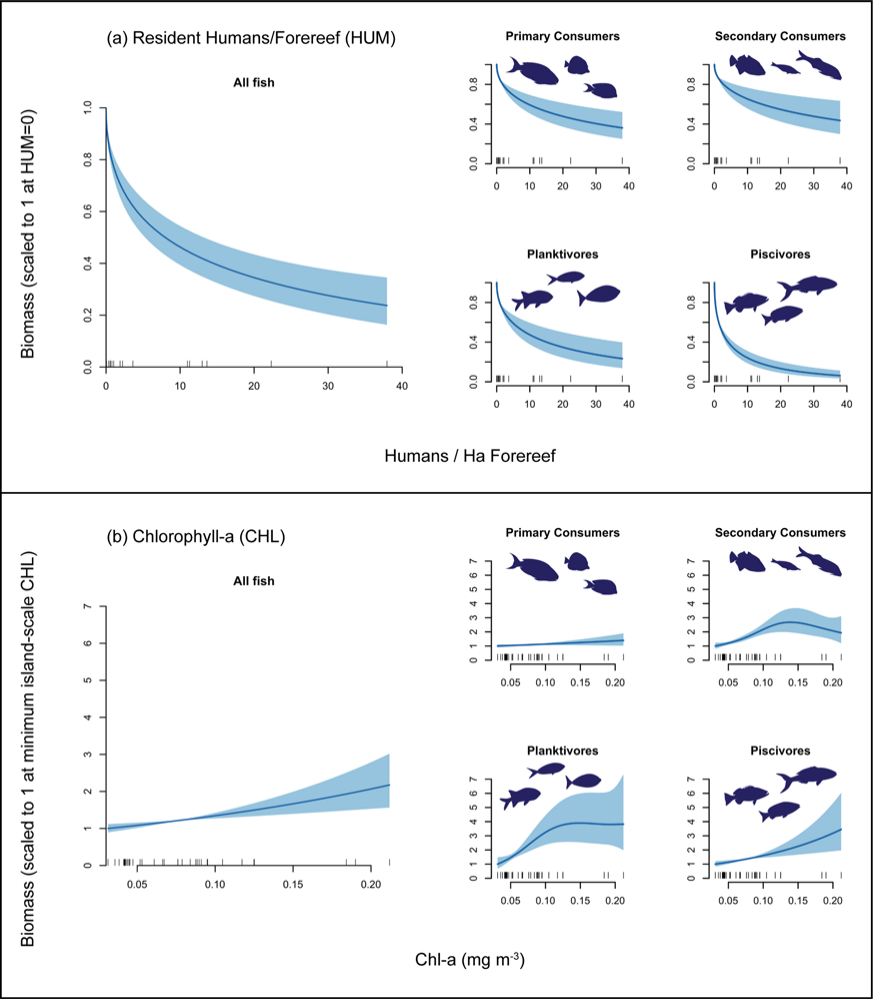
Visualization of smoothers for (a) local humans per area of forereef (HUM) and (b) Chlorophyll-a (CHL). Smoothers were generated using the *predict* function, with data values held at variable means for variables other than the predictor, which was set to values equally spaced between the min and max across all reef areas. Scales of response are shown as proportion of biomass at (i) no humans; and (ii) min CHL.

Retained models of consumer group biomass also indicated strong support for the importance of the two human population predictors: HUM was included in all retained models (vi ≥ 0.998 for all consumer groups, Table 3); and HDIST was included in all retained models for primary consumers, planktivores, and piscivores (vi ≥ 0.920), as well as several of the top-ranked models for secondary consumers (vi = 0.598, Table 3). Visualizations of top-ranked models show rapid declines in reef fish biomass at relatively low levels of HUM followed by more gradual declines as population density increased further (Fig. 3). Biomass declines at low local human population density were particularly steep for piscivores (Fig. 3). Local human population density at the most populous of our study islands, Oahu, corresponded with a decline of >75% of total reef fish biomass, and > 95% of piscivore biomass (Fig. 3).

Chlorophyll-a (CHL) was an important predictor variable not only for all fishes, but also for secondary consumers, planktivores, and piscivores, and was included in all retained models (vi ≥ 0.993 for those groups, Table 3). Evidence for the importance of CHL for primary consumers was more equivocal—it was present in three of the nine retained models, including the top-ranked model (vi = 0.413, Table 3). Visualizations of CHL effects show increases in biomass of all reef fish groups as CHL increased from our island-scale low of 0.03 mg m^-3^ (at Rota) to its high of 0.21 mg m^-3^ (at Jarvis), but the scales of increase varied greatly among groups. For ‘all fishes’, predicted biomass at highest CHL was ~2.2 times that at lowest CHL (Fig. 3). Predicted impacts of elevated CHL were larger for planktivores (highest biomass being ~3.9 times that at lowest CHL) and piscivores (~3.5 times, Fig. 3). In contrast, high CHL was associated with a relatively small change in primary consumers: an ~40% increase in biomass from lowest to highest CHL islands.

Hard coral cover (HC) was an important predictor for primary consumers, being included in all retained models and having variable importance of 0.998 (Table 3), and there was substantial support for its importance to secondary consumers and piscivores (vi = 0.753 and 0.690 respectively), but not for planktivores (vi = 0.090, Table 3, Fig. 2).

Other predictor variables had less consistent effects. One or the other of the two correlated variables SSTL and WV was included in all retained models for planktivores (Table 3, Fig. 2). Whether the reef area was an atoll or not was generally important for planktivores, and to some degree for primary consumers (Table 3). There was some support for a positive relationship between structural complexity (CX) and biomass of primary consumers (included in 4 top-ranked models; vi = 0.585, Table 3), and planktivores (included in the top-ranked model and with variable importance of 0.526, Table 3).

### Model Predictions

**Fig 4:**
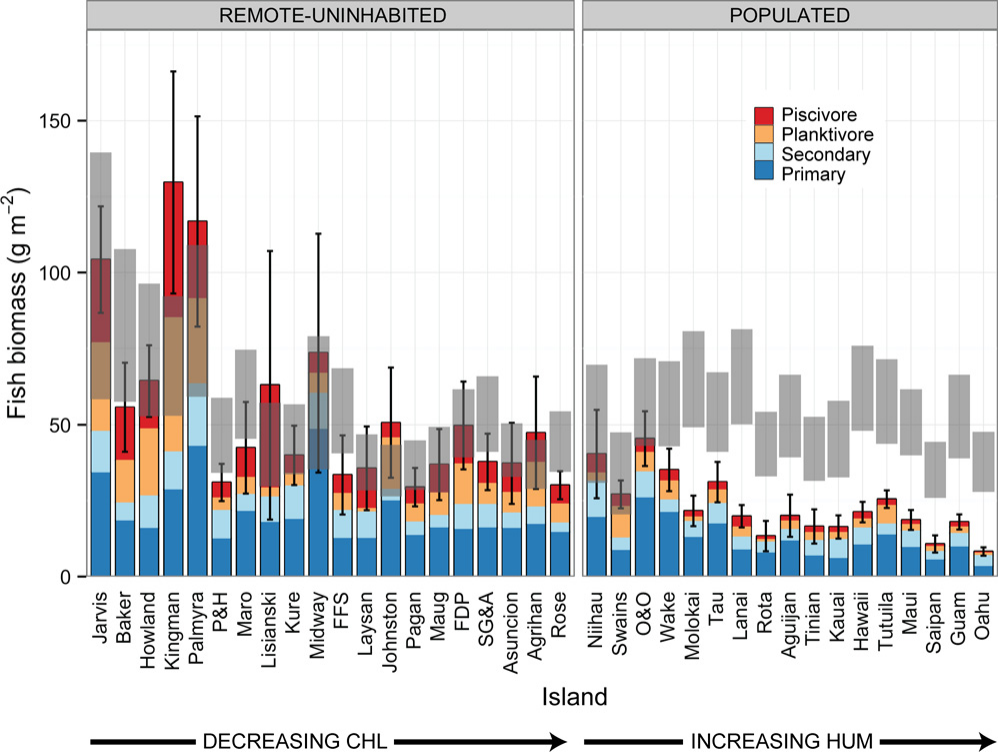
Reef Fish Reference Points generated from model predictions with HSUM and HDIST set to 0. The bars are fish biomass (+95% CI per island). The gray bars are model predictions in absence of humans (low to high CI). Remote and uninhabited islands (left hand panel) are sorted from high to low CHL. Human-populated islands (right hand panel) are sorted from low to high human population density per unit reef area. ‘Remote-uninhabited’ reef areas include places with small resident populations of managers or researchers, but where harvest of reef fishes is prohibited (Midway, Palmyra).

Predicted total reef fish biomass in the absence of people varied by a factor of ~ 3 amongst islands, from a low of 34.0 ± 6.8 g m^-2^ (mean ± SE) at Swains, to a high of 99.0 ± 20.3 g m^-2^ at Jarvis (Fig. 4, S2 Table). By region, predicted total reef fish biomass was highest for the US Line Islands: means of those islands being 72.6 to 99.0 g m^-2^, whereas reef areas in other regions had predicted biomass means of ~35 to ~55–65 g m^-2^ (Fig. 4, S2 Table). Comparison of reef fish biomass estimates derived from visual surveys with predicted biomass in the absence of humans suggests that total reef fish biomass has been depleted by 61% to 69% at populated islands in the Mariana Archipelago; by 20% to 78% in the Main Hawaiian islands (least at Niihau, and most at Oahu); and by 21%, 42% and 56% at, respectively, Ofu & Olosega, Tau, and Tutuila in American Samoa (Fig. 4, S2 Table).

## Discussion

### Impacts of Humans

Over the last decade and more, several studies have highlighted dramatic differences in reef fish assemblages between human-populated and remote coral reef areas, with reef fish biomass at remote reefs typically being several times that at human-populated areas; and with greatest differences for heavily-targeted, larger-bodied and upper trophic level fishes [1–4,31]. Our estimates of depletion at human-populated islands are broadly consistent with those studies. Specifically, at all but the most lightly populated islands, we estimated that reef fish biomass was ~20–40% of what it would be in the absence of humans, and also that human effects were much stronger on piscivores than on other consumer groups. The importance of distant (i.e. non-resident) human population density in nearly all top-ranked models also corroborates recent studies demonstrating that humans have effects on fish assemblages relatively far from human population centers, presumably reflecting the willingness of some fishers to undertake long-range fishing trips [32].

In addition to the several remote islands, inhabited islands in our study ranged from lightly-populated islands, such as Niihau and Ofu & Olosega, to densely populated islands such as Tutuila, Guam and Oahu. Across those inhabited islands, human population density spanned values from <0.02 to nearly 40 people per hectare of forereef habitat. Sharp declines in fish biomass at the low end of that human population scale are consistent with earlier smaller-scale studies on human impacts to coral reef fishes along fishing-intensity and population gradients in Fiji and the Seychelles [33–35]. Precipitous declines in abundance even at low levels of human population density have also been shown for sharks [8], large-bodied excavating parrotfishes [36], and other large fish species [37]. Collectively, there is strong evidence that key aspects of reef fish assemblages including total biomass, top-predator density, and grazing potential, are highly susceptible to even low levels of human impacts, and therefore that full protection over large areas is probably necessary for a natural coral reef ecosystem to persist.

### Influence of Habitat and Environmental Factors

Our study highlights something that is perhaps less widely recognized, namely that there are very substantial differences in reef fish standing biomass among extremely remote coral reef areas where we assume reef fish assemblages are relatively unaffected by human activities. For example, mean reef fish biomass at the US Line Islands (Jarvis, Kingman, Palmyra) averaged nearly two and a half times biomass on reefs in the Northwestern Hawaiian Islands, in spite of the fact that NWHI reefs are extremely distant from the nearest human-population centers (> 600 km). Clearly not all remote coral reefs have the same ability to sustain extremely high reef fish biomass, and, as has been shown for reef sharks [8], a key factor appears to be differences in oceanic productivity among locations. Here we use long-term mean satellite-derived surface chlorophyll-a concentrations (CHL) measured offshore of the 30 m bathymetric contour at each location as our proxy for oceanic productivity. Those values ranged from 0.03–0.21 mg m^-3^ among study areas [7]. When examining the effect of CHL alone, our top-ranked models predicted a > 2-fold increase in total fish biomass across that range, with almost a 4-fold increase in the biomass of planktivores and piscivores.

An obvious explanation for the positive association between oceanic chlorophyll-a and reef fish biomass—particularly of planktivores and piscivores—is that the flux of plankton-rich oceanic waters impinging on and flowing over reefs in highly productive regions sustains large stocks and high productivity of planktivores and presumably also of other components of reef ecosystems such as filter feeders. Increased productivity in those groups in turn supports other components of reef food webs when they are preyed upon, and through defecation and other processes that subsidize reef detrital food webs [38–42]. While it is intuitive that highly productive waters could sustain large populations of planktivores, it is notable that piscivore biomass scaled almost as strongly to CHL. It seems plausible that piscivores benefit from increased productivity of multiple trophic groups. We stress that our models are of reef fish biomass, and patterns of standing fish biomass do not necessarily correspond with what happens to biomass productivity over the same gradients. For example, although reef fish primary producer (i.e. herbivore and detritivore) biomass did not respond particularly strongly to high CHL, it is possible that within-group productivity did, but that much of the excess productivity in areas with high CHL was harvested by abundant local piscivores. More generally, although our large-scale datasets allowed us to identify clear patterns of difference among Pacific reef areas, they do not provide direct information on the processes that drive those differences. There is certainly a need for more research into the physical-biological processes that drive pelagic productivity in the vicinity of reefs and the trophic pathways that link this pelagic productivity with reef fish assemblages.

It has long been recognized that the invertebrate and vertebrate planktivores on reefs consume significant amounts of pelagic plankton, representing large inputs of allochthonous nutrients and organic material into the reef [41,43–46]. It has also been hypothesized that changes in oceanic productivity regimes might lead to changes in reef fish standing stocks over time [47]. However, we are not aware of previous studies that have demonstrated how critically important oceanic productivity may be in regulating island-scale coral reef systems’ capacity to sustain high fish biomass. Recent studies of spatial and temporal patterns of reef shark and monk seal abundance have shown how important it is to incorporate environmental drivers such as oceanic productivity into assessments of human impacts [8,48]. In our study, the apparent impacts of human activities and of oceanic productivity were of broadly the same magnitude. Therefore, we believe it is important that large-scale and long-term studies of factors affecting reef fish assemblages attempt to account for differences in oceanographic setting, particularly productivity, as is routinely done for other marine systems, and for pelagic fisheries [49,50].

Though wave energy, coral cover, and structural complexity did not stand out as important predictor variables in our models of reef fish biomass among islands, these factors can clearly have large impacts on the distribution of local fish biomass [13,14]. Our island-scale results do not cast doubt on the importance of those factors in driving differences in local reef fish assemblages, i.e. among reefs within an island ecosystem, but instead reflect the fact that the absolute scale of differences in those parameters among reefs and reef-zones within islands are potentially much greater than among the island-scale means we used for analysis. However, including those factors in our models removes uncertainty about whether those factors could have been a source of systematic bias in the island-scale patterns we observed among the ~40 Pacific islands and atolls from which we have data.

### Impacts of Survey Methods and Design Choices on Reef Fish Biomass Estimates

Reef fish biomass estimates in our study are considerably lower than those reported for the same islands in a number of earlier studies. For example, mean island-scale biomass values in our study for the uninhabited US Line Islands and the NWHI were ~117 g m^-2^ and ~46 g m^-2^ respectively, around one third to one fifth of the reef fish biomass values reported from those locations in earlier studies [1,2]. There are three main reasons for that. First, as described in the methods section, we excluded sharks and jacks from the analysis because of concerns about possibility of bias caused by different responses of those taxa to presence of divers among study locations [23,24]. Second, we use only ‘instantaneous data’, i.e. snapshot counts based on observation of fishes present within survey cylinders during rapid visual sweeps made by divers in which they attempt to count all fishes of a particular group at one moment in time. The data we use therefore come from ‘closed’ counts; whereas the majority of reef fish visual survey methods, including those utilized in the studies referenced above, are ‘open’ counts, i.e. in which divers record all fishes within or passing into or across a survey area within some period of time, such as in front of a diver as they swim along a transect. While open (non-instantaneous) counts maximize opportunities for gathering data on mobile species—which includes many scarce, roving and heavily targeted taxa of considerable interest to survey and monitoring programs—they will overestimate densities of mobile species, particularly highly mobile groups such as roving piscivores which can make up a considerable portion of reef fish biomass in some remote reef areas [11]. Third, coral reefs are on continuums from well-developed and structurally complex habitats to patchy, low relief, and low coral cover habitats; and low-relief low coral cover habitats typically have much lower reef fish biomass than more complex and more coral-rich habitats [15,51,52]. Therefore, expected coral reef fish biomass depends on what types and condition of coral reef habitats are included within the survey domain. Our surveys were randomly located within a broad domain—all forereef hardbottom in < 30 m deep water. Thus, the data we use are representative of a wide range of reef habitats and depths at each island or atoll, but which include marginal coral habitats that likely have lower fish biomass than areas visited by many coral reef survey programs.

We can quantify the impact of removing counts of sharks and jacks and, to some degree also, of using only instantaneous counts. Including sharks and jacks would have increased US Line Islands mean biomass estimates by ~50% and NWHI biomass estimates by ~ 150%, to 180 and 117 g m^-2^ respectively. As we describe in the methods section, divers recorded some non-instantaneous data during surveys, specifically, counts of fishes of taxa that are observed within an initial 5-minute survey period, but where that species is not present in the survey area at the time of the instantaneous count for that group. We have previously found that the pooled non-instantaneous and instantaneous counts yield similar biomass estimates to the open belt transects used by our program before 2009 [3]. Including non-instantaneous counts would have increased our total biomass estimates for US Line Islands and NWHI by around another 50%, to values of ~249 and 177 g m^-2^; much closer to values for those region reported in earlier studies.

More generally, coral fish survey counts are known to vary depending on multiple factors, including observer swimming speed, survey dimensions, whether a pre-count waiting period is included, and whether a transect line is deployed [53–57]. It is also important to recognize that there are also a number of sources of hard-to-quantify bias, including diver-avoidance by some species, attraction by others, and imperfect detectability [56,58,59] which mean that biomass estimates from visual surveys are best treated as relative rather than absolute measures of density. While we believe our survey approach was suitable for generating comparable estimates of reef fish biomass representative of broad swathes of reef habitat at each reef area, we emphasize that survey methodology and design have large impacts on biomass estimates produced by reef fish surveys, and likely also on the apparent scale of human impacts, as, for example, including non-instantaneous counts of sharks and jacks would have led to much larger biomass disparities between populated and uninhabited islands.

### Conclusions

The availability of large-scale, representative, and highly comparable biological, habitat and oceanographic datasets from 37 reef areas spread widely across the western and central Pacific allowed us to quantify associations between coral reef fish assemblages and a range of human and oceanographic factors, and to use those models to estimate the apparent extent of human impacts while accounting for those other factors. Perhaps the most important component of this study is the demonstration of the extent to which coral reefs’ capacity to support large fish populations varies among what we assume are relatively unimpacted reef areas. In our study, oceanic productivity appeared to be a key driver of those differences, but clearly there are also other factors driving differences among and within island reef ecosystems. We caution against any assumption that the spectacular high biomass fish assemblages seen at some remote reefs represent a natural level that all reefs would attain in the absence of humans.

## Supporting Information

S1 Fig Derivation of substrate height from categorical complexity. Prior to 2011, divers estimated substrate complexity in their stationary point count cylinder on a 5-point scale (1–5). From 2012 onwards, divers estimated proportion of habitat within their survey area in different vertical height bins. In order to utilize substrate data from 2010 and 2011, we calculated the relationship mean vertical height and substrate complexity at the 15 sites where we have both complexity types, and generate a standard conversion formula using linear regression
(TIF)

S1 Table Study islands’ fish & benthic data (mean ± SE). Note sharks and jacks are excluded.
(EPS)

S2 Table Surveyed and predicted reef fish biomass by island. (EPS)

S1 Text R analysis code. (DOCX)
