# Correction: Human, Oceanographic and Habitat Drivers of Central and Western Pacific Coral Reef Fish Assemblages

**DOI:** 10.1371/journal.pone.0129407

**Published:** 2015-05-26

**Authors:** The PLOS ONE Staff

There are formatting errors in Table 3, “Best GAMs (all models with weight > 0.05).” Please see the corrected Table 3 here. The publisher apologizes for the errors.

**Table 3:** Best GAMs (all models with weight > 0.05).

Model Terms									Model Support			
AT	CHL	CX	HC	HDIST	HUM	SSTL^1^	WV^1^	d.f.	Adj-R^2^	AICc	Δ AICc	Weight
ALL FISHES excluding sharks and jacks												
	X		X	X	X			8.5	0.833	288.46	0.00	0.383
X	X		X	X	X			9.3	0.835	290.97	2.52	0.109
	X	X	X	X	X			9.4	0.834	291.19	2.74	0.097
	X		X	X	X	X		11.4	0.866	291.55	3.09	0.081
	X		X	X	X		X	10.0	0.841	292.01	3.55	0.065
**0.212**	**0.990**	**0.256**	**0.852**	**0.993**	**1.000**	**0.219**	**0.114**	←**variable importance sum wt.**→				**0.735**
PRIMARY CONSUMERS												
	X	X	X	X	X			10.6	0.829	224.45	0.00	0.105
		X	X	X	X			10.9	0.834	224.56	0.11	0.099
X		X	X	X	X			10.5	0.826	224.80	0.35	0.088
		X	X	X	X		X	10.7	0.829	224.80	0.35	0.088
X			X	X	X			8.6	0.786	225.04	0.59	0.078
X	X	X	X	X	X			11.3	0.839	225.14	0.69	0.074
			X	X	X		X	8.6	0.784	225.29	0.84	0.069
			X	X	X			7.6	0.762	225.34	0.90	0.067
X	X		X	X	X			9.7	0.805	225.44	0.99	0.064
**0.420**	**0.413**	**0.585**	**0.998**	**0.982**	**0.999**	**0.095**	**0.275**					**0.730**
SECONDARY CONSUMERS												
	X		X	X	X			9.8	0.730	168.39	0.00	0.183
X	X		X	X	X			11.0	0.756	169.66	1.27	0.097
	X		X		X			8.1	0.670	169.68	1.29	0.096
	X				X			4.9	0.552	170.98	2.59	0.050
**0.261**	**0.996**	**0.190**	**0.753**	**0.598**	**0.948**	**0.134**	**0.190**					**0.426**
PLANKTIVORES												
X	X	X		X	X	X		12.3	0.907	180.84	0.00	0.379
	X			X	X	X		10.4	0.879	181.96	1.12	0.217
X	X			X	X	X		11.0	0.885	182.98	2.14	0.130
X	X			X	X		X	9.0	0.854	183.78	2.94	0.087
X	X	X		X	X		X	9.4	0.857	184.53	3.68	0.060
**0.756**	**0.993**	**0.526**	**0.090**	**0.982**	**0.998**	**0.820**	**0.180**					**0.873**
PISCIVORES excluding sharks and jacks												
	X		X	X	X			6.0	0.818	184.11	0.00	0.307
	X		X	X	X	X		7.4	0.824	186.88	2.77	0.077
	X		X	X	X		X	7.3	0.823	187.12	3.01	0.068
	X	X	X	X	X			7.0	0.818	187.17	3.06	0.067
**0.204**	**0.997**	**0.213**	**0.690**	**0.920**	**1.000**	**0.223**	**0.165**					**0.519**

## References

[1] Williams ID, Baum JK, Heenan A, Hanson KM, Nadon MO, Brainard RE. (2015) Human, Oceanographic and Habitat Drivers of Central and Western Pacific Coral Reef Fish Assemblages. PLoS ONE 10(4): e0120516 doi: 10.1371/journal.pone.0120516 25831196 10.1371/journal.pone.0120516 PMC4382026

