# Supplementary figures and images for: Human, Oceanographic and Habitat Drivers of Central and Western Pacific Coral Reef Fish Assemblages
Source: PLoS One. 2015 Apr 1;10(4):e0120516. doi: 10.1371/journal.pone.0120516 (PMC4382026; doi:10.1371/journal.pone.0120516)

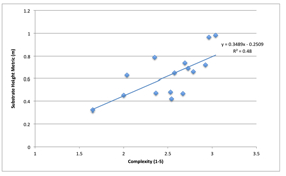

Supplement: S1 Fig — Prior to 2011, divers estimated substrate complexity in their stationary point count cylinder on a 5-point scale (1–5). From 2012 onwards, divers estimated proportion of habitat within their survey area in different vertical height bins. In order to utilize substrate data from 2010 and 2011, we calculated the relationship mean vertical height and substrate complexity at the 15 sites where we have both complexity types, and generate a standard conversion formula using linear regression (TIF) [file pone.0120516.s001.tif]
