# Supplementary material for: Human, Oceanographic and Habitat Drivers of Central and Western Pacific Coral Reef Fish Assemblages
Source: PLoS One. 2015 Apr 1;10(4):e0120516. doi: 10.1371/journal.pone.0120516 (PMC4382026; doi:10.1371/journal.pone.0120516)
Supplement: S1 Text — (DOCX) [file pone.0120516.s004.docx]

## Williams et al. 2015 Human, oceanographic and habitat drivers of Central and Western Pacific Coral Reef Fish Assemblages

========================================================

This is the R code used to run the analysis in the paper.

rm(list=ls()) # clean workspace
library(MuMIn) # needed for model averaging
library(mgcv) # needed for GAM
load(file="WilliamsetalPLosSONE2015.rdata") # reads in dataset z

### Explanation of dataframe variables

- REGION, ISLAND .. region and island names (e.g. "MHI" and"Oahu")
- PRIM Biomass (g/m2) of Primary Consumers
- SECO Biomass (g/m2) of Secondary Consumers
- PLNK Biomass (g/m2) of Planktivores
- PISC_noSJ Biomass (g/m2) of piscivores, excluding sharks and jacks
- ALLF_noSJ Biomass (g/m2) of all fishes, excluding sharks and jacks
- HC Visually estimated Hard Coral Cover (%)
- CX Visually estiamted mean substrate height within survey cylinders (m)
- CHL Oceanic productivity, i.e. long-term mean of satelitte derived Chl-a of waters surrounding the reef areas
- SSTL Climatological low sea surface temperature, satelitte derived
- HUM Square-root transformed human population density per reef
   area (ie humans rsident ont he island divided by area of forereef)
- HDIST Square-root transformed 'distant' human population per reef area (i.e. humans living within 200km of the site but not resident on the island, divided by the area of forereef)
- AT Atoll? (True/False)
- WV Long-term mean estimated island-scale wave energy

### MuMIn routines to generate and rank all possible models, and to model average the selected models

MXPARAMS<-7 # maximum # of parameters in the model
BASE_K<-5 # limit to number of knots

##### Example code for ALLF_noSJ
z$BIO<-z$ALLF_noSJ

#define full model
M1<-gam(BIO ~ s(CX, k=BASE_K)
 + s(CHL, k=BASE_K)
 + s(HC, k=BASE_K)
 + s(HUM, k=BASE_K)
 + s(HDIST, k=BASE_K)
 + s(SSTL, k=BASE_K)
 + s(WV, k=BASE_K)
 + AT,
 data=z, family=Gamma(link=log))

# run all possible combinations, but do not include both SSTL and WV in the same model
options(na.action="na.fail") # prevent fitting models to different datasets

M.set<-dredge(M1,beta=FALSE, rank="AICc", subset=!(`s(SSTL, k = BASE_K)` && `s(WV, k = BASE_K)`), m.max=MXPARAMS, extra = alist(AIC, "R^2", "adjR^2"))

#head(M.set,12) #view top models, and variable importance
#round(importance(M.set),3)

top.models <- get.models(M.set, weight > .05) #select all models with weight > 0.05

# use model averaging of all selected top models
M.avg<-model.avg(top.models) # get averaged coefficients

### Code to generate predictions in absence of humans

A predictor data set is created from the full data set, and set the two human population density variables to 0

PD<-z # duplicate dataset
PD$HUM<-0 # set local human population to 0
PD$HDIST<-0 # set distance human population to 0

# run predict function on the averaged mdoel (generated above)
PRED<-predict(M.avg, newdata=PD, se.fit=TRUE, backtransform=FALSE, type="response")

#generate prediction output data frame
PO<-z[,c("REGION", "ISLAND", "PRIM", "SECO", "PLNK", "PISC_noSJ", "ALLF_noSJ")]
PO$REGION<-as.character(PO$REGION)
PO$fit<-PRED$fit
PO$se<-PRED$se.fit
